# Associations between degree and sub-dimensions of depression and metabolic syndrome (MetS) in the community: results from the Gutenberg Health Study (GHS)

**DOI:** 10.1186/s12888-018-1691-1

**Published:** 2018-04-27

**Authors:** Jörg Wiltink, Matthias Michal, Claus Jünger, Thomas Münzel, Philipp S. Wild, Karl J. Lackner, Maria Blettner, Norbert Pfeiffer, Elmar Brähler, Manfred E. Beutel

**Affiliations:** 1 Department of Psychosomatic Medicine and Psychotherapy, University Medical Center of the Johannes Gutenberg-University Mainz, Mainz, Germany; 2 Center of Cardiology, University Medical Center of the Johannes Gutenberg-University Mainz, Mainz, Germany; 3 Preventive Cardiology and Preventive Medicine, Center for Cardiology, University Medical Center of the Johannes Gutenberg-University Mainz, Mainz, Germany; 4 Center for Thrombosis and Hemostasis, University Medical Center of the Johannes Gutenberg-University Mainz, Mainz, Germany; 5 DZHK (German Center for Cardiovascular Research), partner site RhineMain, Mainz, Germany; 6 Institute of Clinical Chemistry and Laboratory Medicine, University Medical Center of the Johannes Gutenberg-University Mainz, Mainz, Germany; 7 Institute of Medical Biostatistics, Epidemiology and Informatics, University Medical Center of the Johannes Gutenberg-University Mainz, Mainz, Germany; 8 Department of Ophthalmology, University Medical Center of the Johannes Gutenberg-University Mainz, Mainz, Germany

**Keywords:** Metabolic syndrome, Depression, Comorbidity, Somatic, Mental health care

## Abstract

**Background:**

A growing number of studies have associated metabolic syndrome (MetS) and depression, both retrospectively and prospectively. However, it has remained unclear, which degrees, or sub-dimensions of depression are related to MetS and if comorbid depression affects health care utilization. The purpose of the study was to determine the associations of a) somatic and cognitive-affective symptoms to MetS and b) depression and MetS to health care utilization.

**Methods:**

In a population-based, representative survey of 14.499 participants we studied the associations of the two dimensions of depression with MetS and health care utilization. Depressive symptoms were assessed by the Patient Health Questionnaire (PHQ-9).

**Results:**

MetS and its components were associated with the degree of depression, particularly with moderately severe/ severe depressive symptoms (PHQ-9 > = 15). There were clear positive associations of somatic-affective depressive symptoms with the presence of MetS and its components. Cognitive-affective symptoms were negatively associated with MetS. At the single item level, disorders of sleep and appetite as well as exhaustion were positively, while trouble concentrating was negatively associated with MetS. Symptoms of depression were related to higher consultations of somatic and mental health care, while the presence of MetS was related to somatic health care utilization. There was an additional interaction of depressive symptoms and MetS with mental health care.

**Conclusions:**

Somatic affective symptoms of depression are positively associated, while cognitive-affective symptoms are negatively associated with MetS.

## Background

The combination of abdominal obesity, hypertension, dyslipidemia and insulin resistance is termed the metabolic syndrome (MetS). A harmonized definition for MetS proposed in 2009 was used as described in the Methods section [1].

MetS affects about 20% of the general population in western European countries (e.g. [2]). It is clearly associated with cardiovascular diseases and the development of type 2 diabetes (e.g. [3]). Several cross-sectional retrospective studies investigated the relation between depression and MetS by different questionnaires and interviews [4]. Most of the recent studies found significant positive relations between MetS and depression (e.g. [5–7]).

These results are emphasized by a recent meta-analysis of Vancampfort et al. including a total of 18 studies [8].

Prospective studies found evidence for both, MetS increasing the risk for depression as well as depression increasing the risk for MetS, indicating a bidirectional relation between MetS and depression (e.g. [9–12]).

In studies with focus on depression depressive symptoms are assessed by questionnaires e.g. by the PHQ [9] or the CES-D [6, 12] or alternatively by diagnostic interviews [8]. Interestingly, in a substantial amount of studies somatic conditions (e.g. cardiovascular disease, obesity, diabetes) only the somatic-affective and not the cognitive-affective component of depression is related to poor medical outcome [13–17].

There are only few studies differentiating cognitive-affective and somatic-affective depressive symptoms in MetS [18]. To our knowledge, to date there are no studies that have investigated the associations of somatic-affective and cognitive-affective symptoms with MetS in larger population-based samples.

Only few studies differentiated specific depressive symptoms or syndromes and their relation to MetS. In a larger cross-sectional community survey Marijnissen et al. consulted a total of 1.277 participants between 50 and 70 years. All components of MetS were examined and depressive symptoms were assessed with the Beck Depression Inventory (BDI) [18]. MetS as well as the number of metabolic risk factors were significantly related to depressive symptoms (BDI sum score). The associations were primarily driven by somatic-affective symptoms of depression (and not by the cognitive-affective symptoms).

Further attempts were made to cluster depressive symptoms and relate them to MetS.

Takeuchi et al. confirmed the relations between depression and MetS [18, 19]. The authors argued that the main reason for a weakened association between MetS and depression might be caused by ‘typical’ symptoms like decreased appetite or loss of weight, because MetS and one of its core symptoms of central obesity is caused by ‘atypical’ increased appetite or weight gain. In their study with 1.011 male Japanese participants between 20 and 59 years they differentiated ‘atypical depression’ (mainly defined by somatic symptoms like hyperphagia, hypersomnia or leaden paralysis) as a subtype of Major Depressive Disorder (MDD) with atypical features [20] and ‘non-atypical depression’. Only ‘atypical depression’ especially hyperphagia as one of its symptoms was associated with MetS; ‘non-atypical depression’ was not.

In a Finnish population register based study 4.500 randomly-selected men and women aged 45 to 74 years were examined with the BDI [21]. Depressive participants were divided into melancholic (symptoms: e.g. sadness, past failure, loss of pleasure, guilty feelings, punishment feelings, loss of interest) and non-melancholic sub-groups. According to the DSM-IV non-melancholic MDD is defined by atypical depressive symptoms [20] and therefore comparable with the ‘atypical depression’ studied by Takeuchi et al. [18, 19]. One of the main results of the study was that persons with non-melancholic depression more frequently suffered from MetS [21] comparable to the ‘atypical’ depressed reported by [19].

In our cross-sectional population based sample we sought to answer the following questions:

1. Are intensity and symptomatology of depression (somatic-affective and/or cognitive-affective) associated to metabolic syndrome (MetS)?
2. How are depressive symptoms and metabolic syndrome (MetS) associated to the utilization of mental and somatic health care?

We hypothesized a closer relation between MetS and somatic-affective depressive symptoms compared to cognitive-affective symptoms. Moreover, we hypothesized a more frequent health care utilization in participants suffering from both depressive symptoms and MetS.

## Methods

### Procedure and study sample

A total of *N* = 14.499 participants were enrolled in the Gutenberg Health Study (GHS) between the years 2007 and 2012 and investigated cross-sectionally. The Gutenberg Health Study is a German population-based, prospective, observational single-center cohort study in the Rhine-Main-Region. To date a total of 15.010 participants have been included. The primary aim of the study is to analyze and improve cardiovascular risk factors and their stratification. The local ethics committee and the local and federal data safety commissioners have approved the study procedure. The participants were determined randomly from the local registry of the city of Mainz and of the district of Mainz-Bingen. The sample was stratified for sex, residence and age. Inclusion criteria were written informed consent and age between 35 and 74 years. Persons with insufficient German language knowledge were not included in the study, as well as persons who were not able to come to the study center due to physical and/or mental impairment. The response rate was 60.3% for the first 5.000 participants of the study; it was determined as the recruitment efficacy proportion. A detailed description of the design and the rationale of the GHS have been published elsewhere [22].

### Materials and assessment

Participants were examined in a 5-h baseline assessment in the study center of the GHS. The examination comprised investigation of cardiovascular risk factors, clinical and laboratory parameters (from venous blood), blood pressure, anthropometric measurements and a computer-assisted interview. All the tests were conducted fulfilling standard procedures by certified staff.

### Primary outcome measures

#### Depression

Symptoms of depression were assessed with the German version of the Patient Health Questionnaire (PHQ-9 [23]). Caseness was defined by a score ≥ 10. Löwe et al. found a sensitivity of 81% and a specificity of 82% for depressive disorder determined by this cut-off [23]. We followed Kroenke et al. regarding their classification of depressive symptoms: “minimal” (score 5 to 9), “mild” (score 10 to 14), “moderately severe” (score 15 to 19) and “severe” (score > 20) [24]. The two dimensions of depressive symptoms (somatic-affective and cognitive-affective) were defined according to earlier studies [25–27]. Four items of the PHQ-9 related to problems with appetite, sleep, psychomotor agitation/retardation, and fatigability. They were classified as somatic-affective symptoms. Five items related to lack of depressed mood, interest, concentration problems, negative feelings about self, and suicidal ideation. Those items were classified as cognitive-affective symptoms [14, 16, 17]. There are a considerable number of studies assessing the factor structure of the PHQ-9 in the general population. Some studies repeatedly found a single factor of depression (e.g. [28, 29]), while others favored a two factorial model (e.g. [30, 31]).

We were aware, that dimensions of depression (cognitive-affective and somatic-affective) in community samples (e.g. [31]) might differ from the dimensions from cardiovascular settings (e.g. [25–27]). However, for comparison purposes and due to their face validity and comparability we used these dimensions.

#### Metabolic syndrome

For the definition of metabolic syndrome we followed the harmonized recommendations of Alberti et al. [1].

Participants have to fulfil at least 3 of the following criteria: (1) triglycerides > = 150 mg/dl or on drug treatment for increased triglycerides; (2) high-density lipoprotein cholesterol (HDL) < 40 mg/dl in men and < 50 mg/dl in women or on drug treatment for reduced high-density lipoprotein cholesterol; (3) blood pressure increased to > 130 mmHg systolic or > 85 mmHg diastolic or on antihypertensive drug treatment; (4) Obesity: waist circumference; > = 80 cm for female and > =95 cm for male participants (5) fasting blood glucose > = 100 mg/dl or on drug treatment for increased glucose.

#### Health care utilization

Health care utilization was assessed by self-report. Participants were asked whether they had consulted general practitioners or medical specialists (somatic physicians) and/or psychotherapists/ psychiatrists during the last month.

### Potential confounders

A set of confounding variables potentially related to MetS and/or depression (in addition to sex and age) was predefined.

#### Life style factors

*The socioeconomic status* (SES) was defined according to Lampert’s and Kroll’s scores of SES ranging from 3 to 21 while 3 indicates the lowest SES and 21 the highest SES [32]. The score is multidimensional including information on school-based and occupational education, occupational position and income.

#### Anxiety

*We assessed generalized anxiety* symptoms with the short form (two items) of the GAD-7 (Generalized Anxiety Disorder [GAD] – 7 Scale) [33, 34]. A sum score of 3 or more (range 0–6) indicates generalized anxiety with good sensitivity (86%) and good specificity (83%) [34]. Symptoms of *panic* were screened with the brief panic module of the PHQ. We defined caseness if at least two (of the first four) panic items are answered with “yes” [35]. The Mini-Social Phobia Inventory (Mini-Spin) [36, 37] was used to detect symptoms of *social anxiety*. A cut-off score of 6 (range 0–12) separates between persons with generalized social anxiety disorder and controls with good sensitivity (89%) and specificity (90%) [36, 38]. *Any anxiety* was defined by reaching the cut-off in one or more of the three mentioned scales [38].

#### Somatic conditions

*Diabetes* was defined in individuals with a definite diagnosis of diabetes by a physician or HbA1c > 6.5% or drug treatment for diabetes. *Obesity* was defined as a Body-Mass-Index (BMI) ≥30 kg/m^2^. The presence of further somatic conditions was assessed within a structured interview: “Has a physician ever diagnosed: constriction of your coronary heart vessels (*coronary heart disease,* CHD), *atrial fibrillation*, *cancer*, *myocardial infarction*, *stroke, or peripheral arterial occlusive disease (PAOD)*?”

#### Psychotropic medication

*Psychotropic medications* potentially affecting mood and/ or metabolism were selected as confounders: antidepressants (selective serotonin reuptake inhibitor, non-selective monoamine reuptake inhibitors, and others), anxiolytics, hypnotics/ sedatives, antipsychotics, opioids, antiepileptics.

### Statistical analysis

Statistical analysis was done by IBM SPSS Statistics 20 (IBM, Chicago, IL).

Data on depressive symptoms (PHQ-9) and MetS were essential for the analyses in this paper. Therefore, we omitted subjects with missing data in at least one of these two conditions from further analyses. For 249 participants the MetS status could not be obtained; in 239 of these participants fasting blood glucose was not available, the remaining 11 participants had other missings. Due to missings in PHQ-9 the sum score could not be computed for 278 cases. Overall 511 participants of the GHS could not be included into the analyses because of missing data (PHQ or MetS).

Odds ratios of single items differentiating the population with and without MetS were computed by logistic regression analyses of the dichotomized PHQ-9 items (0 = “not at all” vs. 1 = “several days”, 2 = “more than half the days”, 3 = “nearly every day”) on MetS status. The models were adjusted by age, sex and SES.

Data are presented as numbers/percentage, mean (and 1.96-fold standard deviation) or median (and 1st, 3rd quartile) as appropriate.

For the analyses of the relationship between depressive symptoms and MetS, depressive symptoms and the number of risk determinants of MetS and depressive symptoms and the five risk determinants of MetS, we calculated separate linear regression models with depressive symptoms (PHQ-9 sum score) as dependent variable. Model 1 was not adjusted; model 2 was adjusted for age, sex and socioeconomic status (SES) as potential confounders of depressive symptoms and MetS (and the number of risk determinants of MetS). Model 3 was additionally adjusted for somatic conditions (CHD, atrial fibrillation, cancer, myocardial infarction, stroke, PAOD), any anxiety, and psychotropic medication. Depressive symptoms were examined with two separate analyses using somatic-affective and cognitive-affective symptoms of depression as dependent variables. In model 4 analyses were additionally adjusted for cognitive-affective symptoms when the somatic affective component was the dependent variable and for somatic-affective symptoms when the cognitive affective component was the dependent variable. PHQ-9 data were skewed distributed. Therefore, we transformed the dependent variables to improve the regression model: ln(PHQ-9 sum score + 5), ln(somatic-affective sum score + 5), ln(cognitive-affective sum score + 2). In this context ln denotes the natural logarithm. Thus, the range of the variables and the impact of outliers have been reduced. By including the natural logarithmic variables not only linear but also non-linear association can be statistically proven.

Relations between depression determined by PHQ (sum score < 10 vs. sum score > = 10), MetS and health care utilization were determined by using logistic regression. In these analyses we used *consultation of somatic physicians* (yes/ no) and b) *consultation of psychotherapists/ psychiatrists* (yes/ no) as dependent variables. MetS, depression and their interaction term (MetS × depression) were the independent variables in this model. We controlled for age, sex, socioeconomic status (SES), any anxiety and somatic conditions (myocardial infarction, CHD, stroke, PAOD, cancer, and atrial fibrillation).

All *p*-values correspond to 2-tailed tests. The ascertained p-values are interpreted descriptively in terms of an explorative data analysis.

## Results

### Sample characteristics

In Table 1 we show the sample characteristics. The data were stratified for severity of depressive symptoms.

**Table 1:** Sample characteristics stratified for severity of depressive symptoms (*N* = 14.499)

	Depressive symptoms (PHQ score)			
	0–4	5–9	10–14	15–27
	no	minimal	mild	moderately severe/severe
Number	9403 ^a^	3977	846	273
Sex, female	45.3%	56.7%	59.8%	61.2%
Age, in years; mean (SD)	55.2 (11.2)	54.4 (11.0)	53.1 (10.3)	51.7 (9.8)
SES, (3–21), median (1st/ 3rd quartile)	13.0 (10/17))	12.0 (9/16)	11.0 (9/15)	11.0 (8/14)
Current smoker	17.9%	21.0%	27.1%	30.1%
Depressive symptoms				
Somatic-affective, median (1st/ 3rd quartile)	1 (1/2)	4 (3/4)	6 (5/7)	9 (7/10)
Cognitive-affective, median (1st,/3rd quartile)	0 (0/1)	3 (2/4)	5 (4/6)	9 (8/10)
Any anxiety	3.7%	19.5%	56.4%	86.4%
Psychopharmacological treatment	4.9%	12.4%	24.6%	43.2%
Somatic conditions				
Risk determinants of MetS median (1st/3rd quartile)	2 (1/3)	2 (1/3)	2 (1/3)	2 (1/3)
Metabolic syndrome (full criteria)	32.0%	32.7%	34.4%	41.8%
High triglycerides(> = 150 mg/dl or on drug treatment for increased triglycerides)	23.8%	25.4%	26.8%	33.0%
Low HDL (< 40 mg/dl in men and < 50 mg/dl in women)	16.9%	18.6%	27.1%	25.5%
Hypertension(> 130 mmHg systolic or > 85 mmHg diastolic or on antihypertensive drug treatment)	65.3%	62.9%	61.1%	65.3%
Central obesity(waist circumference > 88 cm in women and > 94 cm in men)	67.0%	70.4%	74.3%	78.0%
High fasting blood glucose(> = 100 mg/dl or on drug treatment for increased glucose)	21.8%	19.5%	18.5%	17.2%
Diabetes	8.5%	9.8%	11.5%	11.8%
Obesity (BMI > =30)	23.0%	26.9%	30.5%	38.6%
Atrial fibrillation	2.6%	2.8%	2.4%	3.3%
CHD	3.9%	4.7%	5.4%	5.9%
Myocardial infarction	2.6%	3.5%	2.7%	3.3%
PAOD	2.8%	4.0%	5.1%	6.4%
Stroke	1.6%	1.9%	3.0%	1.8%
Cancer	8.5%	10.1%	10.2%	7.0%
Health care utilization (last 4 weeks)				
Consulted somatic physicians	39.3%	46.8%	57.1%	64.0%
Consulted psychiatrists	0.2%	0.5%	2.1%	9.2%
Consulted psychotherapists	0.4%	0.9%	3.7%	8.1%

^a^ A total of 511 participants were excluded from analyses because of missing data: 278 participants had missings in PHQ-9; 249 participants had missings in parameters necessary for the determination of MetS, mostly fasting blood glucose in 238 participants

A total of 65% reported no, 27% minimal, 6% mild, and 2% moderately severe and severe depressive symptoms.

With an increasing severity of depressive symptoms, there was an increase of the proportion of females and a decrease of mean age. The proportion of current smokers increased, along with psychopharmacological treatments. The proportions of a metabolic syndrome increased only slightly from no (32%) to minimal and more from mild to moderate/ severe depressive symptoms (42%). Similar increases were found for the components of metabolic syndrome (esp. lipids, central obesity, but not hypertension, resp. fasting glucose). However, there was an increase of diabetes, obesity, atrial fibrillation, CHD, and PAOD. Consultations of somatic physicians, psychiatrists and psychotherapists also increased with the increasing severity of depressive symptoms.

### Associations between depressive symptoms and MetS

In the linear regression models (model 2), the number of risk determinants of MetS was positively correlated with depressive symptoms after adjustment by age, sex, socioeconomic status (SES) and also after additionally controlling for somatic conditions (model 3). When we used the somatic-affective symptoms of depression as dependent variable and adjusted by the cognitive-affective component, results were similar (model 4). After controlling for age, sex, socioeconomic status (SES) and somatic-affective symptoms cognitive-affective symptoms of depression were negatively correlated with the number of risk determinants of MetS (model 4). See Table 2.

**Table 2:** Linear regression analyses of MetS (number of risk determinants) and depression (*N* = 14.416–14.499 ^a^)

	Depressive symptoms (PHQ)	Somatic-affective symptoms	Cognitive-affective symptoms
	B (95% CI)	B (95% CI)	B (95% CI)
Model 1	0.005 (0.000/ 0.009) *p* = .036	0.010 (0.006/ 0.013) *p* < .001	−0.008 (− 0.013/ -0.002) *p* = .010
Model 2	0.014 (0.010/ 0.019) *p* < .001	0.017 (0.013/ 0.020) *p* < .001	0.002 (−0.004/ 0.008) *p* = .498
Model 3	0.008 (0.003/ 0.012) *p* = .001	0.012 (0.009/ 0.015) *p* < .001	−0.006 (− 0.011/ 0.000) *p* < .053
Model 4	–	0.013 (0.010/ 0.016) *p* < .001	−0.016 (− 0.021/ -0.011) *p* < .001

Model 1: without adjustment; Model 2: adjusted for age, sex and socioeconomic status (SES); Model 3: additionally adjusted for somatic conditions (CHD, atrial fibrillation, cancer, myocardial infarction, stroke, PAOD), any anxiety, and psychotropic medication; Model 4: additionally adjusted for cognitive-affective symptoms (dependent variable: somatic-affective symptoms), respectively for somatic-affective symptoms (dependent variable: cognitive-affective symptoms)

^a^*N* ranges between 13.363 and 14.499 because of missing data

The above mentioned results were more pronounced using the binary variable MetS (fulfilling vs. not fulfilling criteria) as independent variable. See Table 3.

**Table 3:** Linear regression analyses of MetS and depression (*N* = 14.416–14.499 ^a^)

	Depressive symptoms (PHQ)	Somatic-affective symptoms	Cognitive-affective symptoms
	B (95% CI)	B (95% CI)	B (95% CI)
Model 1	0.008 (− 0.004/ 0.020) *p* = .188	0.020 (0.011/ 0.028) *p* < .001	−0.020 (− 0.036/ -0.005) *p* = .011
Model 2	0.028 (0.016/ 0.041) *p* < .001	0.034 (0.025/ 0.043) *p* < .001	0.000 (− 0.016/ 0.016) *p* = .995
Model 3	0.014 (0.003/ 0.026) *p* < .015	0.024 (0.015/ 0.032) *p* < .001	−0.015 (− 0.030/ 0.000) *p* = .056
Model 4	–	0.026 (0.018/ 0.033) *p* < .001	−0.037 (− 0.050/ -0.023) *p* < .001

Model 1: without adjustment; Model 2: adjusted for age, sex and socioeconomic status (SES); Model 3: additionally adjusted for somatic conditions (CHD, atrial fibrillation, cancer, myocardial infarction, stroke, PAOD), any anxiety, and psychotropic medication; Model 4: additionally adjusted for cognitive-affective symptoms (dependent variable: somatic-affective symptoms), respectively for somatic-affective symptoms (dependent variable: cognitive-affective symptoms)

^a^*N* ranges between 13.363 and 14.499 because of missing data

Analyzing the relation of the five components of MetS to depressive symptoms after controlling for age, sex, socioeconomic status (SES) there was a strong positive relation between depressive symptoms and high triglyceride, low HDL and central obesity. In this model (model 2) the relation of depressive symptoms to hypertension and high fasting blood glucose was somewhat weaker and negative. The same was true for additional adjustment for somatic conditions (model 3).

Using only the somatic-affective and cognitive-affective symptoms of depression as dependent variables (model 4) hypertension and high fasting blood glucose were unrelated to somatic-affective and negatively related to cognitive-affective symptoms. Central obesity had the strongest relation to somatic-affective symptoms of depression.

A similar picture emerged for model 4, additionally controlled for cognitive-affective resp. somatic-affective symptoms. See Table 4.

**Table 4:** Linear regression analyses of the five components of MetS and depression (*N* = 14.148–14.496 ^a^)

		Depressive symptoms (PHQ)	Somatic-affective symptoms	Cognitive-affective symptoms
		B (95% CI)	B (95% CI)	B (95% CI)
Model 1	High triglycerides Low HDL Hypertension Central obesity High fasting blood glucose	0.020 (0.007/ 0.034); *p* = .003 0.050 (0.036/ 0.065); *p* < .001 −0.028 (− 0.040/ -0.016); *p* < .001 0.044 (0.031/ 0.056); *p* < .001 − 0.039 (− 0.053/ -0.024); *p* < .001	0.019 (0.010/ 0.029); *p* < .001 0.042 (0.031/ 0.053); *p* < .001 − 0.007 (− 0.016/ 0.001); *p* = .094 0.048 (0.039/ 0.057); *p* < .001 0.005 (− 0.025/ -0.005); *p* = .004	0.011 (−0.006/ 0.028); *p* = .219 0.040 (0.020/ 0.059); *p* < .001 − 0.051 (− 0.066/ -0.036); *p* < .001 0.008 (− 0.007/ 0.024); *p* = .296 − 0.065 (− 0.083/ -0.046); *p* < .001
Model 2	High triglycerides Low HDL Hypertension Central obesity High fasting blood glucose	0.026 (0.011/ 0.040); *p* < .001 0.033 (0.017/ 0.049); *p* < .001 − 0.008 (− 0.022/ 0.005); *p* = .211 0.035 (0.022/ 0.048); *p* < .001 − 0.022 (− 0.037/ -0.007); *p* = .003	0.017 (0.007/ 0.028); *p* = .001 0.027 (0.016/ 0.038); *p* < .001 0.002 (− 0.008/ 0.011); *p* = .739 0.038 (0.028/ 0.047); *p* < .001 − 0.009 (− 0.019/ 0.002); *p* = .117	0.028 (0.009/ 0.047); *p* = .004 0.028 (0.007/ 0.048); *p* = .009 − 0.021 (− 0.038/ -0.003); *p* = .019 0.008 (− 0.010/ 0.025); *p* = .385 − 0.039 (− 0.058/ -0.019); *p* < .001
Model 3	High triglycerides Low HDL Hypertension Central obesity High fasting blood glucose	0.019 (0.006/ 0.032); *p* = .005 0.020 (0.005/ 0.034); *p* = .008 − 0.012 (− 0.025/ 0.000); *p* = .043 0.024 (0.013/ 0.036); *p* < .001 − 0.020 (− 0.034/ -0.006); *p* = .004	0.013 (0.003/ 0.023); *p* = .011 0.017 (0.007/ 0.028); *p* = .002 0.000 (− 0.009/ 0.009); *p* = .985 0.031 (0.022/ 0.040); *p* < .001 − 0.008 (− 0.018/ 0.002); *p* = .128	0.020 (0.002/ 0.038); *p* = .026 0.014 (− 0.005/ 0.033); *p* = .155 − 0.028 (− 0.044/ -0.012); *p* = .001 − 0.004 (− 0.020/ 0.012); *p* = .611 − 0.036 (− 0.054/ -0.018); *p* < .001
Model 4	High triglycerides Low HDL Hypertension Central obesity High fasting blood glucose		0.008 (−0.001/ 0.016); *p* = .090 0.012 (0.003/ 0.022); *p* = .010 0.007 (− 0.001/ 0.015); *p* = .080 0.031 (0.024/ 0.039); *p* < .001 0.000 (− 0.009/ 0.009); *p* = 1.000	0.009 (− 0.007/ 0.024); *p* = .267 − 0.002 (− 0.019/ 0.015); *p* = .810 − 0.029 (− 0.043/ -0.015); *p* < .001 − 0.030 (− 0.044/ -0.017); *p* < .001 − 0.029 (− 0.045/ -0.013); *p* < .001

Model 1: without adjustment; Model 2: adjusted for age, sex and socioeconomic status (SES); Model 3: additionally adjusted for somatic conditions (CHD, atrial fibrillation, cancer, myocardial infarction, stroke, PAOD), any anxiety, and psychotropic medication; Model 4: additionally adjusted for cognitive-affective symptoms (dependent variable: somatic-affective symptoms), respectively for somatic-affective symptoms (dependent variable: cognitive-affective symptoms)

^a^*N* ranges between 13.363 and 14.499 because of missing data

Participants with MetS scored higher than those without MetS in the following three items: “Trouble falling or staying asleep, or sleeping too much” (item 3; OR 1.11), “Feeling tired or having little energy” (item 4; OR 1.13), and “Poor appetite or overeating” (item 5; OR 1.45).

The three items all belong to the somatic-affective symptoms of depression.

Participants with MetS scored lower in the item “Trouble concentrating on things, such as reading the newspaper or watching television” (item 7; OR 0.88). This item belongs to the cognitive-affective symptoms of depression (Table 5).

### Relation between depressive symptoms, MetS and health care utilization

The relationship between depressive symptoms (cut-off > = 10), MetS and health care utilization was examined by logistic regressions.

Depressive symptoms were related to the consultations of psychotherapists/psychiatrists (OR 3.51 [95%CI 0.43/ 1.19], *p* < .001) after adjustment (by age, sex, SES, anxiety, somatic conditions), and so was the interaction between depressive symptoms and MetS (OR 2.66 [95%CI 1.34/5.26], *p* = .005). MetS alone was not related to consultations of psychotherapists/psychiatrists (OR 0.72 [95%CI 0.43/1.19], *p* = .202).

Again, after controlling for the aforementioned variables (age, sex, SES, anxiety, and somatic conditions) depressive symptoms (OR 1.63 [95%CI 1.37/1.94], *p* < .001) and MetS (OR 1.15 [95%CI 1.06/1.25], *p* < .001) were significantly related to consultations of somatic physicians, the interaction between depressive symptoms and MetS (OR 1.12 [95%CI 0.84/1.25], *p* = 0.451) was not.

## Discussion

The principal findings of this paper are a) a significant relation between MetS and depressive symptoms; b) somatic-affective symptoms of depression were positively, whereas cognitive-affective symptoms are negatively associated with MetS, c) depressive symptoms and MetS were both independently related to somatic health care utilisation.

MetS and its components increased with the degree of depression, particularly with moderately severe/severe depressive symptoms (PHQ-9 > = 15). As we had hypothesized, there were clear positive associations of somatic-affective symptoms with the presence of MetS and with its components. However, cognitive-affective symptoms were negatively associated with MetS. At the single item level, disorders of “sleep” and “appetite” as well as “exhaustion” were positively, while “trouble concentrating” was negatively associated with MetS. Thus, the association of depressive symptoms and MetS was primary based on somatic-affective symptoms. The negative relation of MetS to cognitive-affective symptoms was somewhat unexpected. However, comparing the PHQ-9 items that defined cognitive- affective depressive symptoms (lack of interest, depressed mood, negative feelings about self, concentration problems and suicidal ideation) had substantial overlap with the symptoms defining melancholic depression by BDI (e.g. sadness, past failure, loss of pleasure, guilty feelings, punishment feelings, loss of interest) used in other studies (e.g. [21]). This overlap might be the reason for similar results: lower prevalence of MetS in the subsample of participants with melancholic depression than in the subsample of non-melancholic depressed participants in the Study of Seppälä et al. [21] compared to the negative relation between cognitive-affective symptoms and MetS in our study.

Examining the relations of the five components of MetS (increased triglycerides, decreased HDL, increased blood pressure, obesity, increased fasting blood glucose) to depressive symptoms a strong negative relation between the severity of cognitive-affective symptoms and hypertension as well as fasting blood glucose were responsible for the relation between MetS and cognitive-affective symptoms (see Table 4). A negative relation of systolic blood pressure (SPB) and cognitive-affective symptoms has already been reported by Michal et al. for participants free of antihypertensive drugs [39]. The results also correspond to findings of other studies [40, 41]. Several explanations for a relation between depressive symptoms and SPB have been hypothesized. For example chronic low blood pressure might cause depression through somatic symptoms and fatigue, however longitudinal studies found heterogeneous results on this relationship [41]. Furthermore, it is speculated that the central monoamine system with altered levels of neuropeptide Y – suppressing sympathetic activity and thus decreasing blood pressure and increasing depressive symptoms - might contribute to this relationship (e.g. [39, 41–43]). The neuroendocrine mechanisms likely involved are only partly understood [40].

**Table 5:** Depressive Symptoms (PHQ): Comparison between participants with (*N* = 4.717) and without MetS (*N* = 9.782)

	OR (95%CI)
1 - Little interest or pleasure in doing things	1.01 (0.94/ 1.09)
2 - Feeling down, depressed, or hopeless	1.06 (0.98/ 1.15)
3 - Trouble falling or staying asleep, or sleeping too much	**1.11** (1.03/ 1.20)
4 - Feeling tired or having little energy	**1.13** (1.04/ 1.22)
5 - Poor appetite or overeating	**1.45** (1.34/ 1.57)
6 - Feeling bad about yourself or that you are a failure or have let yourself or your family down	0.96 (0.88/ 1.05)
7 - Trouble concentrating on things, such as reading the newspaper or watching television	**0.88** (0.82/ 0.95)
8 - Moving or speaking so slowly that other people could have noticed. Or the opposite being so fidgety or restless that you have been moving around a lot more than usual	1.00 (0.90/ 1.11)
9 - Thoughts that you would be better off dead, or of hurting yourself	1.09 (0.96/ 1.25)

Logistic regression analyses of PHQ-9 items on MetS, models adjusted for age, sex and SES. Odds ratio above 1 indicate a trend towards a higher item response in MetS. ORs and 95%CI are presented; categories of the PHQ-9 items were dichotomized: 1–3 (several days, more than half the days, nearly every day) vs. 0 (not at all)

Bold *p* < .001 are statistically significant

It is known from the literature that cognitive processes are directly affected by blood glucose levels (e.g. [44]). Gradual depletion of blood glucose causes fatigue and a decline in cognitive function [45]. Thus it is no surprise that cognitive-affective symptoms like “trouble concentrating” are negatively related to fasting blood glucose as a relevant component of MetS.

As expected depressive symptoms were related to higher consultations of both somatic and mental health care, while the presence of MetS was related to a higher somatic health care utilization. For patients suffering from both MetS and depressive symptoms only mental health care utilization was increased (not somatic health care utilization). A reason for this somewhat surprising finding might be the more delibitating character of depressive symptoms compared to MetS causing more consultations of health care professionals. However, health care utilization was self-reported and limited to the past 4 weeks, which might affect the reliability (e.g. recall-bias).

The study is limited due to the cross-sectional data acquisition. Thus, causal interpretations of the results are impossible. Furthermore, our results might be limited regarding persons with severe depression because of a potential selection bias might towards oversampling participants with less severe depressive symptoms. Further limitation is evident by the use of data from validated self-rating scales. However, we could not use clinical expert ratings of depression.

While we could study a very large and population-based sample, interpretation is limited by the retrospective nature of the survey [17]. Furthermore, we excluded persons with insufficient language knowledge and those who were not able to visit the study center on their own which might also pertain to the generalizability of the results.

Beside the mentioned limitations of our study there are several strengths: a) the well characterized and representative sample of persons living in the Rhine-Main region of Germany b) the inclusion of younger participants starting with the age of 35 years and c) the large sample size.

Future, prospective analyses of the sample will determine the potential bidirectional nature of the relationship, i.e. not only if depression predicts future development of MetS, but also, if the presence of MetS or its components predicts the occurrence of depressive symptoms.

## Conclusions

Somatic affective symptoms of depression are positively associated with MetS, while cognitive-affective symptoms are negatively associated with MetS.

## Abbreviations

BDI: Beck Depression Inventory
BMI: Body-Mass-Index
CES-D: Center for Epidemiological Studies-Depression
CHD: Coronary heart disease
CI: Confidence interval
DSM-IV: Diagnostic and statistical manual of mental disorders, 4th edition
GAD-7: Generalized Anxiety Disorder Scale
GHS: Gutenberg Health Study
HbA1c: Hemoglobine A1c
HDL: High-density lipoprotein cholesterol
Ln: Natural logarithm
MDD: Major Depressive Disorder
MetS: Metabolic syndrome
Mini-Spin: Mini-Social Phobia Inventory
N: Number
OR: Odds ratio
PAOD: Peripheral arterial occlusive disease
PHQ-9: Patient Health Questionnaire, depression module
SES: Socioeconomic status
SPB: Systolic blood pressure
SPSS: Statistical Package for the Social Sciences
